# Associations Between Air Pollution Exposure and Gestational Weight Gain Pattern: Evidence from a Large-Scale Hospital-Based Retrospective Cohort Study

**DOI:** 10.3390/toxics14030264

**Published:** 2026-03-18

**Authors:** Shimin Xiong, Wenting Ai, Kunming Tian, Xiaoming Zhu, Man Chen, Xubo Shen, Boyi Yang, Yuanzhong Zhou

**Affiliations:** 1 School of Public Health, Zunyi Medical University, Zunyi 563000, China; xiongshimin@zmu.edu.cn (S.X.); wentingai615@163.com (W.A.);; 2 Key Laboratory of Maternal and Child Health and Exposure Science, Guizhou Provincial Department of Education, Zunyi 563000, China; 3 Guiyang Maternal and Child Health Hospital Guiyang Children’s Hospital, Guiyang 550002, China; 4 Department of Occupational and Environmental Health, School of Public Health, Sun Yat-Sen University, Guangzhou 510080, China

**Keywords:** air pollution, gestational weight gain, retrospective cohort, epidemiology

## Abstract

Air pollution has been associated with dysregulated metabolism. However, evidence linking prenatal air pollution exposure to gestational weight gain (GWG) pattern remains limited. This retrospective cohort study of 47,793 pregnant women in Guiyang (2013–2022) assessed associations between air pollutants and GWG pattern. Positive associations were observed between excessive GWG and CO (per 1 μg/m^3^ increase), NO_2_, O_3_, PM_10_, PM_2.5_, and SO_2_ (per 10 μg/m^3^ increase) throughout the whole pregnancy period. Specifically, early-pregnancy exposure to CO (OR = 1.377, 95% CI: 1.201, 1.578) and NO_2_ (OR = 1.098, 95% CI: 1.068, 1.130), along with exposure to PM_10_ (OR = 1.058, 95% CI: 1.043, 1.073), PM_2.5_ (OR = 1.095, 95% CI: 1.073, 1.118), and SO_2_ (OR = 1.135, 95% CI: 1.102, 1.169) during late pregnancy significantly increased excessive GWG risk. Conversely, O_3_ exposure was inversely associated with excessive GWG. For insufficient GWG, only early-pregnancy exposures to PM_10_ (OR = 1.016, 95% CI: 1.001, 1.032), PM_2.5_ (OR = 1.022, 95% CI: 1.001, 1.043), and SO_2_ (OR = 1.031, 95% CI: 1.004, 1.058) showed significant positive associations. Furthermore, the restricted cubic spline (RCS) model revealed a nonlinear relationship between pollutant exposure and the risk of excessive GWG. Stratified analyses revealed that the air pollution and GWG (continuous) association was stronger among women with pre-pregnancy BMI ≥ 24 kg/m^2^ and aged ≥ 30 years. This study confirms that, even at lower concentrations, exposure to air pollutants during pregnancy is significantly associated with an increased risk of abnormal GWG. Compared to previous studies focusing on high-concentration areas, this finding provides additional evidence for assessing the health risks of air pollution exposure during pregnancy, suggesting that the potential metabolic effects of low-level, long-term exposure should be considered when developing maternal health strategies.

## 1. Introduction

Gestational weight gain (GWG) is a physiologic adaptation essential for successful pregnancy and fetal development. However, both insufficient and excessive GWG are associated with adverse outcomes for mothers and newborns [1]. Insufficient GWG increases the risks of miscarriage, preterm birth, and low birth weight [1,2,3]. Similarly, excessive GWG also raises the risks of gestational hypertension, diabetes, and cesarean delivery [4,5,6]. More importantly, offspring delivered by excessive GWG women face a higher long-term risk of obesity [7]. Therefore, GWG is not only a key indicator of maternal health during pregnancy, but also a comprehensive reflection of metabolic adaptation during gestation, holding significant clinical and public health implications.

Air pollution is a global public health concern that poses serious threats to maternal and fetal health. Long-term exposure to air pollution is associated with higher incidences of pregnancy complications, including anemia, hypertension, and diabetes [8,9,10]. In addition, air pollution exposure is also a recognized risk factor for dysregulated metabolism, including obesity, overweight, and metabolic diseases, among the general population [11,12,13]. Notably, the obesogenic effect derived from air pollution is observed across different age groups [14,15].

Long-term cumulative exposure to air pollution during pregnancy could lead to metabolic dysregulation, increasing susceptibility to circadian disruption, similar to the synergistic effect observed in assisted reproductive technology (ART) and Gestational diabetes mellitus (GDM) interactions [16]. While pregnancy itself causes profound metabolic remodeling to support fetal development, these factors may synergistically interfere with energy metabolism, thereby affecting GWG. However, epidemiological studies investigating the association between air pollution exposure and GWG pattern, especially for insufficient GWG, are scarce. One prior cohort study in Wuhan, China, reported that higher prenatal PM_2.5_ exposure was associated with an increased risk of GWG [17]. Another study linked a composite environmental risk factor (encompassing high air pollution, poor food environments, and low green space) to an increased risk of excessive GWG [18]. However, the detrimental health effects of air pollution were mainly explored in higher air pollution contexts, and evidence from low-pollution areas is lacking. Currently, research on the relationship between air pollution and GWG pattern warrants further investigation, particularly for insufficient GWG.

Guiyang, located on the eastern Yungui Plateau, is characterized by karst topography and high vegetation coverage. These geographical features contribute to good air quality, with 98.9% of days meeting or exceeding the national air quality standards. The disturbed metabolic effects generated by lower pollution levels remain to be investigated. In the present study, we aim to detect the relationship between exposure to low levels of air pollutants and GWG pattern (including excessive GWG and insufficient GWG) based on low-air-pollution exposure population, and to explore the possible modifications of pre-pregnancy BMI (ppBMI) and age. Our work provides new epidemiological evidence from low-air-pollution setting on the perinatal GWG pattern.

## 2. Materials and Methods

### 2.1. Study Population

**Figure 1:**
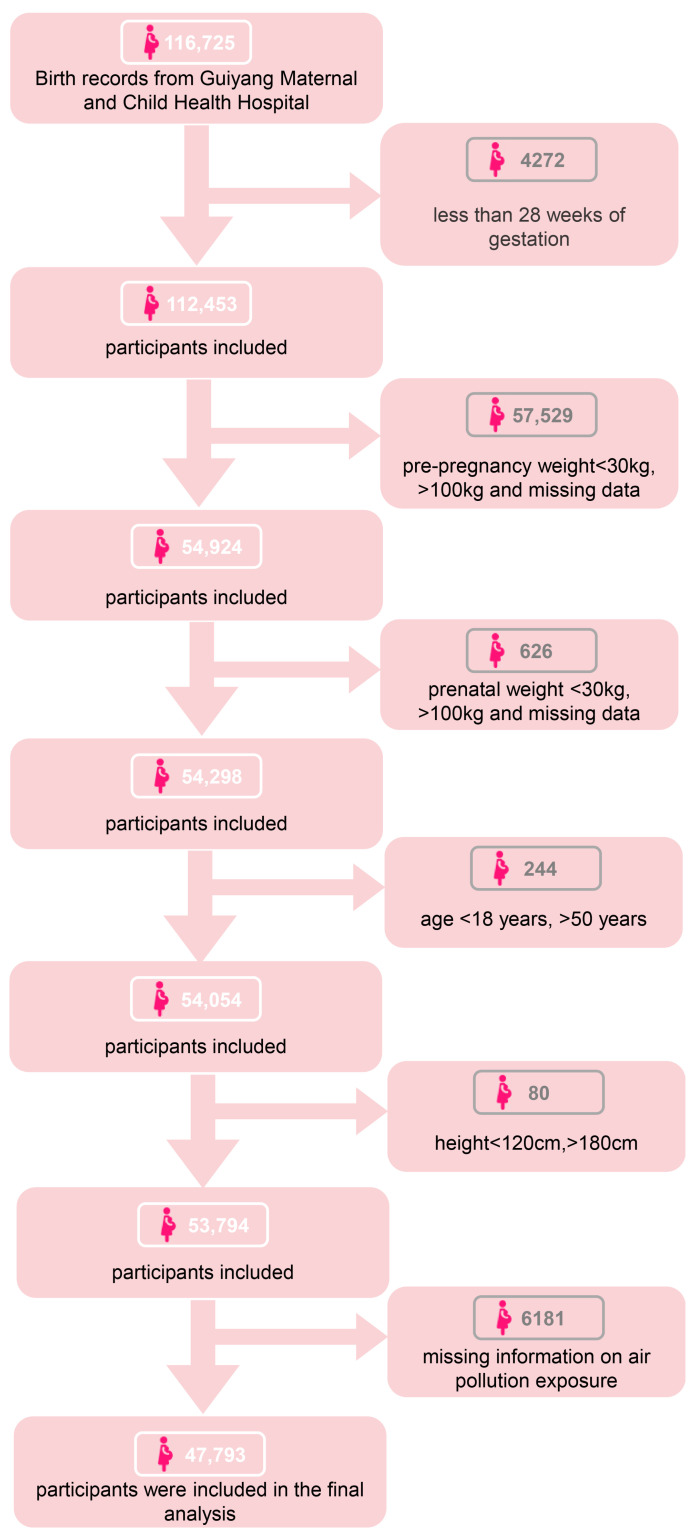
Flow diagram of participant selection.

This study initially identified 116,725 participants from Guiyang Maternal and Child Health Hospital between 2013 and 2022. As shown in Figure 1, we excluded 4272 women with a gestational age of less than 28 weeks, resulting in 112,453 live singletons. We then restricted the analysis to individuals with complete pre-pregnancy and antenatal weight records, leaving 54,298 participants. Subsequently, we applied restrictions for maternal age (18–50 years) and height (120–180 cm), leaving 53,794 participants. Finally, we excluded 6181 participants with missing data on air pollution exposure. This study protocol was approved by the Ethics Committee of Zunyi Medical University (batch No. [2019] H-005).

### 2.2. Exposure Assessment

Six air pollutants, CO, NO_2_, O_3_, PM_10_, PM_2.5_, and SO_2_, were considered, using data from the: China High Air Pollutants (CHAP) dataset. The CHAP dataset provides high-resolution, reliable air pollution estimates by integrating ground-based observations and remote sensing satellite data through comprehensive analyses that incorporate meteorological data, soil moisture, snow cover, and other geophysical parameters. Monthly concentration data were obtained from the CHAP dataset and were spatially matched to each participant’s residential address to estimate monthly exposure concentrations. Trimester-specific exposure was calculated as the average concentration across the three months constituting each trimester. It should be noted that this study used the permanent address at the time of enrollment as the basis for exposure assessment throughout the entire pregnancy, and information on residential mobility during pregnancy was not available. If residential changes occurred, estimated exposure concentrations may not fully represent actual individual exposure levels.

### 2.3. Outcome Definition

Between 6 and 12 gestational weeks of early pregnancy, women came to the hospital for antenatal visits and establish their first electronic medical records. Data on maternal characteristics (sociodemographic, residence, and medical histories) were abstracted from this medical record. Weight in this medical record was also used as the pre-pregnancy weight, and to calculate ppBMI, due to the potential bias of self-reported pre-pregnancy weight. Women were asked to fast in the morning, and weight in both early and late pregnancy were measured by the standard digital scale. Late pregnancy weight was measured upon their admission to the hospital. Research has shown that early pregnancy weight is very close to pre-pregnancy weight and is commonly used as a reliable substitute in pregnancy cohorts [19,20,21]. GWG was calculated as the difference between the last measured weight before delivery and the first recorded early-pregnancy weight, in kilograms. According to IOM guidelines, women were categorized by ppBMI as underweight (<18.5 kg/m^2^), normal weight (18.5–24.9 kg/m^2^), overweight (25.0–29.9 kg/m^2^), or obese (≥30.0 kg/m^2^). The corresponding recommended total GWG ranges are 12.5–18 kg, 11.5–16 kg, 7–11.5 kg, and 5–9 kg, respectively [22]. Based on these ranges, GWG pattern was classified as insufficient, adequate, or excessive.

### 2.4. Covariates

Covariates were selected a priori based on the previous literature [17,23]. These included maternal age (continuous), ppBMI (continuous), ethnicity (Han or minority), education level (≤high school or >high school), gestational age (continuous), parity (multiparous or nulliparous), fetal number (singleton or multiple), infant birth weight (continuous), infant sex (male or female), history of hypertensive disorders or gestational diabetes (yes or no), smoking status (ever or never), alcohol use (ever or never), self-reported nutritional status during pregnancy (good or poor), and season of delivery (spring, summer, autumn, winter). Additionally, we adjusted for ambient temperature and relative humidity (continuous) during pregnancy.

### 2.5. Statistical Analysis

Descriptive statistics are presented as means ± standard deviations for continuous variables and as frequencies (percentages) for categorical variables. Single-pollutant multiple linear regression models were used to assess the associations between per 1 μg/m^3^ increase in CO and per 10 μg/m^3^ increase in NO_2_, O_3_, PM_10_, PM_2.5_, and SO_2_ exposure, and GWG (continuous). Binary logistic regression models were used to estimate odds ratios (ORs) and 95% confidence interval (CI) for the risk of excessive GWG or insufficient GWG associated with per 1 μg/m^3^ increase in CO and per 10 μg/m^3^ increase in NO_2_, O_3_, PM_10_, PM_2.5_, and SO_2_ exposure. Moreover, weighted air pollution scores (APSs) were constructed to assess the combined effects of five pollutants (PM_2.5_, PM_10_, NO_2_, SO_2_, O_3_) on GWG (continuous) and abnormal GWG patterns. The formula of APS was: Air Pollution Score = (β(PM_2.5_) × PM_2.5_ + β(PM_10_) × PM_10_ + β(NO_2_) × NO_2_ + β(SO_2_) × SO_2_ + β(O_3_) × O_3_)/(5/sum of β coefficients) [24]. Furthermore, generalized estimating equations (GEEs) were employed to further examine the relationships between air pollutant exposure of the whole pregnancy period and the risks of insufficient or excessive GWG and GWG (continuous). We also applied RCS to explore their nonlinear relationships. Model was fully adjusted for maternal age, ppBMI, ethnicity, mother’s education, gestational week, litter size, fetal category, infant weight, infant sex, history of hypertensive disorders or gestational diabetes mellitus, smoking status, alcohol use, season of delivery, comorbidities, and ambient temperature and humidity during pregnancy. We further determined the potential effect modifications by ppBMI (normal, overweight/obese, and underweight) and maternal age (<30 years and ≥30 years) on the above associations. We also performed three sensitivity analyses to evaluate the robustness of our findings. First, we excluded women who delivered at gestational weeks 28 to 34. Second, we excluded participants with stillbirth, neonatal death, induced labor, or multiple pregnancies, due to the potential influences of these maternal complications on the above observed associations. Finally, to avoid over-adjusting for infant birth weight, gestational age, and pregnancy complications, we constructed a simplified model excluding these variables to further validate the robustness of our findings. All analysis was completed by R (version 4.3.2, R Foundation for Statistical Computing, Vienna, Austria) and SPSS (version 29.0, IBM Corp., Armonk, NY, USA).

## 3. Results

### 3.1. Baseline of Population

The demographic characteristics of the final cohort, comprising 47,793 women, were summarized in Table 1. The mean maternal age was 29.42 ± 4.97 years. The majority of participants were of Han ethnicity (77.1%), had an education level of high school or above (69.4%), had singleton pregnancies (97.0%), and were first-time mothers (48.0%); few had gestational diabetes (10.5%) or gestational hypertension (0.20%); most were non-smokers (92.9%) and non-drinkers (93.0%). Based on IOM guidelines, most women had normal pre-pregnancy BMI (71.0%), while 39.2% experienced excessive GWG, and 22.0% had insufficient GWG. In addition, the distribution of individual air pollutant concentrations across pregnancy trimesters are presented in Figure S1.

### 3.2. Association Between Air Pollution and GWG Pattern

Initially, we determined the associations between trimester-specific air pollution exposure and GWG (modeled as continuous), presented in Table 2. Exposure to CO, NO_2_, PM_10_, PM_2.5_, and SO_2_ throughout pregnancy was positively associated with GWG. Conversely, O_3_ exposure was negatively associated with GWG in the first (β = −0.099, 95% CI: −0.141, −0.056), second (β = −0.267, 95% CI: −0.307, −0.227), and third trimesters (β = −0.090, 95% CI: −0.132, −0.048).

**Figure 2:**
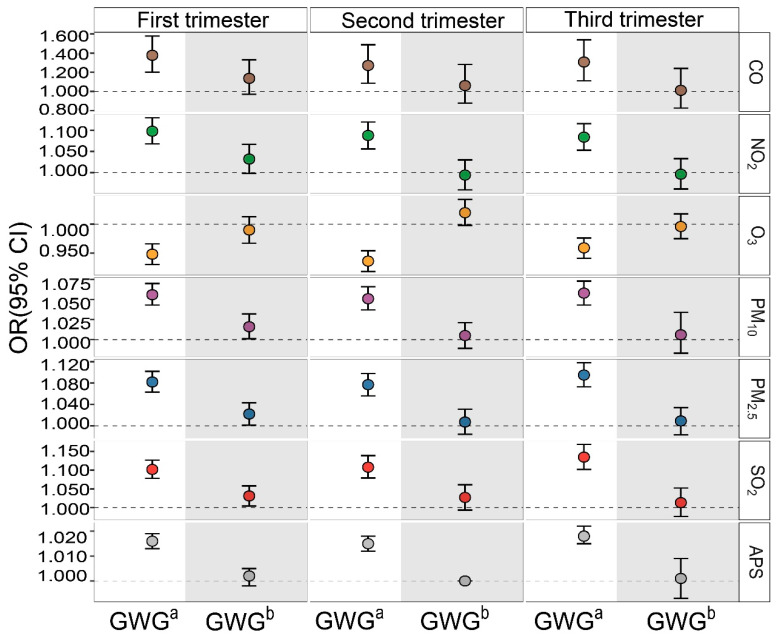
Associations between exposure to pollutants (per 1 μg/m^3^ for CO; and per 10 μg/m^3^ for NO_2_, O_3_, PM_10_, PM_2.5_, SO_2_) and GWG pattern (kg). GWG ^a^: Excessive weight gain during pregnancy, using appropriate weight gain during pregnancy as a control. GWG ^b^: Insufficient weight gain during pregnancy, using appropriate weight gain during pregnancy as a control. APS: Air Pollution Score. First trimester: early pregnancy; Second trimester: mid-pregnancy; Third trimester: late pregnancy. The model was adjusted for maternal age, ppBMI, ethnicity, mother’s education, gestational week, litter size, fetal category, infant weight, infant sex, history of hypertensive disorders or gestational diabetes mellitus, smoking status, alcohol use, season of delivery, comorbidities, and ambient temperature and humidity during pregnancy.

We further determined the associations between air pollution exposure and the risk of abnormal GWG pattern in Figure 2. Specifically (Table S1), early-pregnancy exposure to CO (OR = 1.377, 95% CI: 1.201, 1.578) and NO_2_ (OR = 1.098, 95% CI: 1.068, 1.130), as well as late-pregnancy exposure to PM_10_ (OR = 1.058, 95% CI: 1.043, 1.073), PM_2.5_ (OR = 1.095, 95% CI: 1.073, 1.118), and SO_2_ (OR = 1.135, 95% CI: 1.102, 1.169) were significantly associated with an increased risk of excessive GWG. Conversely, O_3_ exposure during pregnancy was associated with a decreased excessive GWG risk, particularly during the third trimester (OR = 0.959, 95% CI: 0.941, 0.976). For insufficient GWG, significant associations were confined to early-pregnancy exposure to PM_10_ (OR = 1.016, 95% CI: 1.001, 1.032), PM_2.5_ (OR = 1.022, 95% CI: 1.001, 1.043), and SO_2_ (OR = 1.031, 95% CI: 1.004, 1.058). Collectively, perinatal exposure to even low-level air pollution correlated with dysregulated GWG pattern.

We further evaluated the association of APS, which reflects the combined air pollution effect, with GWG. As shown in Table 2 and Table S1, a higher APS was positively associated with excessive GWG risk. This association was more pronounced in late pregnancy (β = 0.005, 95% CI: 0.004, 0.006). Subsequently, we employed GEE to fitted whole-pregnancy-period air pollution exposure to investigate the associations between air pollution and insufficient or excessive GWG and GWG (modeled as continuous). Significant associations of CO, NO_2_, PM_10_, PM_2.5_, and SO_2_ exposure with excessive GWG (Table 3) were consistently found. Among these pollutants, CO exhibited the strongest association (OR = 1.273, 95% CI: 1.113, 1.456). Additionally, exposure to O_3_, PM_10_, PM_2.5_, and SO_2_ was significantly associated with insufficient GWG. Notably, SO_2_ exhibited a relatively strong association with insufficient GWG (OR = 1.050, 95% CI: 1.024, 1.077). We subsequently conducted sensitivity analyses by excluding participants with a gestational age of less than 34 weeks, as well as those with stillbirths, miscarriages, or multiple pregnancies, and further omitted infant birth weight, gestational age, and pregnancy complications. The significance and direction of the observed associations remained largely unchanged (Tables S4–S9).

### 3.3. The Nonlinear Associations Between Air Pollutions and GWG

**Figure 3:**
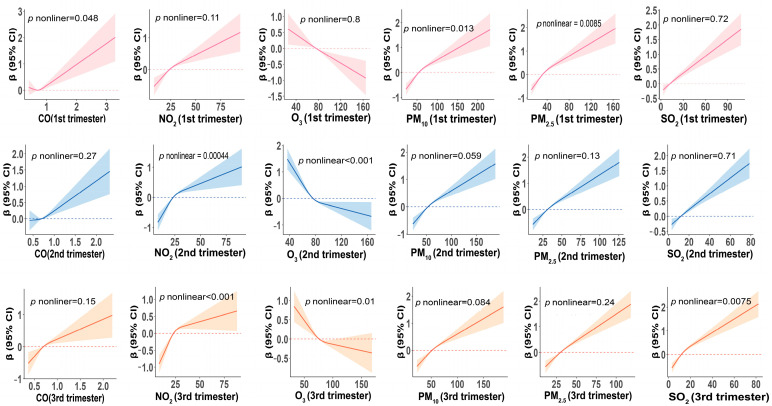
Restricted cubic spline curves were used to analyze the association between air pollution (μg/m^3^) and GWG (modeled as continuous unit: kg). The model was adjusted for maternal age, ppBMI, ethnicity, mother’s education, gestational week, litter size, fetal category, infant weight, infant sex, history of hypertensive disorders or gestational diabetes mellitus, smoking status, alcohol use, season of delivery, comorbidities, and ambient temperature and humidity during pregnancy. Abbreviations: 1st trimester, early pregnancy; 2nd trimester, mid-pregnancy; 3rd trimester, late pregnancy.

We examined the nonlinear relationships between each pollutant and insufficient or excessive GWG and GWG (modeled as continuous) (Figure 3, Figures S2 and S3). This study found that exposure to CO and PM_10_ during early pregnancy, as well as exposure to NO_2_ and O_3_ during mid- and late pregnancy, was significantly associated with total GWG (continuous) in a nonlinear manner (all *p*-nonlinear < 0.05). Air pollution exposure during other stages did not exhibit significant nonlinear association with total GWG (continuous) (all *p*-nonlinear > 0.05).

### 3.4. Stratified Analysis

Stratified analysis revealed that the associations between each trimester exposure to air pollutants with GWG (modeled as continuous) were more pronounced among pregnant women with a BMI ≥ 24 kg/m^2^ and aged ≥ 30 years (Tables S2 and S3). Specifically, significant interactions with ppBMI were found for PM_10_ exposure during early and mid-pregnancy, as well as exposure to PM_2.5_ exposure throughout the entire pregnancy (*p*-interaction < 0.05). These associations were more pronounced among women with a ppBMI ≥ 24 kg/m^2^. Additionally, this study found that exposure to PM_10_ and PM_2.5_ during early and mid-pregnancy, as well as exposure to SO_2_ during early pregnancy, were significantly associated with maternal age (*p*-interaction < 0.05). These associations were more pronounced among women aged ≥ 30 years.

## 4. Discussion

Our study demonstrates that perinatal exposure to relatively low levels of air pollutants is associated with abnormal GWG patterns. Exposure to CO, NO_2_, PM_10_, PM_2.5_, and SO_2_ was associated with an elevated risk of excessive GWG. Notably, for insufficient GWG, significant associations were confined to the first trimester of pregnancy, with PM_10_, PM_2_._5_ and SO_2_ increasing the risk. These findings contribute to deepening understanding of the disturbed metabolic shift among pregnant women associated with low-level air pollution, particularly for air pollution-associated insufficient GWG.

Cumulative evidence confirmed air pollution exposure’s positive association with obesity risk [25]. However, most epidemiological studies mainly focused on air pollution-generated health effects on children and adults, with limited research focus their detrimental health effects on pregnant women. Previous evidence found that higher prenatal PM_2.5_ exposure was associated with increased GWG [17]. Another study further confirmed that exposure to high levels of air pollution (PM_2.5_, PM_10_, and SO_2_) is associated with risk of excessive GWG [18]. Furthermore, a prospective cohort study assessing monthly PM_2.5_ exposure in early and late pregnancy found that association with total GWG varied by the timing of exposure and ppBMI [23]. Compared to studies examining the association between air pollution exposure and GWG, our study employed relatively low exposure levels. For example, a study conducted in Wuhan, China, reported pollutant concentrations (CO, NO_2_, O_3_, PM_10_, PM_2.5_, SO_2_) of 24.3 μg/m^3^, 55.0 μg/m^3^, 52.0 μg/m^3^, 65.8 μg/m^3^, 68.5 μg/m^3^, and 9.7 μg/m^3^, respectively, whereas our study measured pollutant concentrations (CO, NO_2_, O_3_, PM_10_, PM_2.5_, SO_2_) at 0.7 μg/m^3^, 23.7 μg/m^3^, 76.0 μg/m^3^, 55.4 μg/m^3^, 31.8 μg/m^3^, and 12.2 μg/m^3^, respectively [18]. In another comparable study, the average exposure level to PM_2.5_ was approximately three times that observed in this study [17]. Our work strengthens the obesogenic effect of air pollution across the whole pregnancy, albeit at lower exposure concentrations located in developing areas. Notably, we firstly found that mid-pregnancy NO_2_ exposure increased insufficient GWG risk, while early- and mid-pregnancy O_3_ decreases insufficient GWG risk.

Increasing evidence suggests that inflammation and oxidative stress induced by air pollution may represent key mediating pathways linking exposure to abnormal weight gain during pregnancy [26]. Upon inhalation, ambient pollutants can trigger an imbalance in the maternal redox state, characterized by an overproduction of reactive oxygen species (ROS). This overwhelms endogenous antioxidant defenses, such as the thioredoxin reductase 1 (TXNRD1) system, and can initiate a cycle of cellular damage, including mitochondrial DNA injury and subsequent mitochondrial dysfunction [27]. The resultant amplification of oxidative and nitrative stress cascades into the activation of pro-inflammatory transcription factors like NF-κB, leading to the sustained elevation of inflammatory cytokines and the establishment of a chronic, low-grade inflammatory microenvironment [26,27,28,29]. This pollution-driven pro-inflammatory state could have multiple implications for the components of GWG. Beyond its systemic effects—which have been linked in both animal models and human studies to metabolic dysfunctions such as insulin resistance—this inflammatory milieu may also influence placental function. For instance, it could interfere with the processes of trophoblast invasion and spiral artery remodeling, potentially impairing placental perfusion and, thus, affecting the delivery of oxygen and nutrients to the developing fetus [30,31,32]. Such alterations would primarily impact the fetal and placental components of GWG. Simultaneously, heightened systemic inflammation in the pregnant woman herself is a known correlate of accelerated or excessive GWG [23], suggesting a possible link to the maternal component. However, given that GWG is a composite proxy for maternal metabolic adaptation, rather than a direct measure of pathology, further studies are needed to disentangle the specific contributions of each pathway to the overall weight gain pattern.

Interestingly, this study found that total ozone exposure during pregnancy was negatively correlated with gestational weight gain, consistent with the conclusions of several previous studies [18,33]. We propose several possible explanations for this association. First, ozone may influence appetite and energy balance by disrupting the leptin signaling pathway, thereby inhibiting fetal growth and subsequently leading to reduced gestational weight gain [18,34]. Second, O_3_ concentrations often show negative correlations with primary pollutants like NO_2_, suggesting that elevated O_3_ levels may indirectly reflect lower exposure to other positively correlated pollutants (e.g., traffic-based particulate matter), thereby manifesting as a negative association with GWG. Additionally, studies suggest that low-dose O_3_ exposure may induce adaptive responses, activating Nrf2-mediated antioxidant pathways to enhance cellular antioxidant capacity and potentially regulate energy metabolism processes [35,36]. Finally, meteorological factors (e.g., temperature and daylight duration) may exert dual effects on O_3_ formation and weight-related behaviors in pregnant women (e.g., outdoor activity, dietary habits), leaving residual confounding unresolved [37,38]. Future studies employing multi-pollutant models and personal exposure monitoring are needed to clarify these complex relationships.

Our study has several strengths. First, the large, retrospective cohort design enhances the statistical power and generalizability of our findings. Second, we simultaneously considered a range of air pollutants, including CO, NO_2_, O_3_, PM_10_, PM_2.5_, and SO_2_. The integrated APS allowed us to evaluate the combined effect of multiple air pollutants. Third, our study was conducted in a region with relatively good air quality, providing unique insights into the health effects of lower-level pollution. Furthermore, we investigated the associations of air pollution with both excessive GWG and insufficient GWG, whereas previous research has primarily focused on excessive GWG. Finally, we additionally adjusted for ambient temperature and relative humidity, important potential confounders that are often unavailable.

Several limitations should be considered. First, while we adjusted for a range of covariates, important lifestyle factors during pregnancy, such as physical activity, dietary patterns, and screen time were not included, due to their lack of availability in electronic records. Future studies should employ a prospective cohort design, incorporating wearable devices and standardized dietary questionnaires, to more accurately assess the independent effects and interactions of these variables on GWG. Second, exposure was estimated based on residential addresses at enrollment, and residential mobility during pregnancy was not accounted for, which could lead to exposure misclassification, although some studies suggest that residential mobility may not substantially influence air pollution estimates [39]. Additionally, we lacked data on time–activity patterns and occupational exposures, which also contribute to differences between personal and ambient exposure levels. Furthermore, the relatively low and temporally stable air pollution levels in Guizhou may limit the generalizability of our findings to populations experiencing higher or more variable exposure. The study sample size was significantly reduced from the initial cohort, primarily due to missing weight records and air pollution exposure data. This potential selection bias may limit the representativeness of the study sample. Although we included all individuals with complete data to maximize the use of available information, caution is warranted when extrapolating these findings to other populations. Future studies should strive to improve data collection to reduce missingness, or employ methods such as multiple imputation to address missing data and mitigate selection bias. Furthermore, this study did not account for factors such as indoor exposure, daily outdoor activities, or occupational exposure, which may have contributed to discrepancies between estimated and actual exposure levels. Future studies should address these limitations by collecting potential confounding factors through more detailed questionnaires and, where feasible, employing personal exposure monitoring. Finally, data on trimester-specific weight gain were not available, meaning we could not determine how trimester-specific exposures influenced the GWG pattern.

## 5. Conclusions

In summary, our study provides evidence that exposure to CO, NO_2_, PM_10_, PM_2.5_, and SO_2_ is associated with an increased risk of excessive GWG. Moreover, we found that exposure to PM_10_, PM_2.5_, and SO_2_ during early pregnancy was significantly associated with insufficient GWG. Additionally, the association between air pollution exposure and total GWG was more pronounced among women with ppBMI ≥ 24 kg/m^2^ and aged ≥ 30 years. This study provides novel epidemiological evidence in a low-exposure setting, reinforcing the association between air pollution and abnormal patterns of gestational weight gain. Although the impact of air pollution exposure on individual gestational weight gain appears minimal, abnormal gestational weight gain remains a persistent risk factor for multiple adverse pregnancy outcomes. Even small changes could lead to hundreds of additional adverse pregnancy outcomes. Therefore, investigating the association between low-level air pollution exposure and pregnancy weight gain holds significant public health implications. Further validation across diverse populations and in-depth exploration of underlying biological mechanisms are warranted. Future research should also focus on the long-term effects of abnormal pregnancy weight gain on maternal and infant health, including its potential role in pregnancy complications, postpartum weight retention, and offspring metabolic and neurodevelopmental outcomes, to provide a more comprehensive basis for clinical interventions and public health strategies.

## Figures and Tables

**Table 1:** Basic characteristics of the study population (*N* = 47,793).

Variable			*n*	Mean ± SD or Percent
Age			47,793	29.42 ± 4.973
Pre-pregnancy BMI, kg/m^2^				
		<18.5	8342	17.50
		18.5–24.9	33,943	71.00
		25–29.9	4796	10.00
		≥30	712	1.50
Race				
		Han	36,870	77.10
		Minorities	10,923	22.90
Education level				
	High school degree or below		14,567	30.50
	College degree or above		33,161	69.40
	Missing		65	0.10
Smoke				
		Yes	215	0.40
		No	44,397	92.90
		Missing	3181	6.70
Drink				
		Yes	125	0.30
		No	44,456	93.00
		Missing	3212	6.70
Parity				
		Yes	22,960	48.00
		No	24,833	52.00
Multiparity				
		1	42,341	97.00
		≥2	1297	3.00
Neonatal sex				
		Male	25,417	46.80
		Female	22,365	53.20
Complications				
		Yes	40,324	84.40
		No	7464	15.6
		Missing	5	0.00
Season of conception				
		Spring	11,576	24.20
		Summer	12,699	26.60
		Fall	11,615	24.30
		Winter	11,903	24.90
GWG category according to IOM				
		Insufficient GWG	10,491	22.00
		Adequate GWG	18,549	38.80
		Excessive GWG	18,753	39.20
Gestational hypertension				
		Yes	215	0.20
		No	44,397	99.80
Gestational diabetes				
		Yes	5265	10.50
		No	42,523	89.50
		Missing	5	0.00

Abbreviations: GWG: gestational weight gain, IOM: International Organization of Medicine.

**Table 2:** Associations between exposure to pollutants (per 1 μg/m^3^ for CO; and per 10 μg/m^3^ for NO_2_, O_3_, PM_10_, PM_2.5_, SO_2_) and GWG.

Outcome	Exposure	First Trimester		Second Trimester		Third Trimester	
		β (95%CI)	*p*	β (95%CI)	*p*	β (95%CI)	*p*
GWG (kg)							
	CO	0.624 (0.312, 0.935)	<0.001	1.204 (0.812, 1.596)	<0.001	0.836 (0.454, 1.217)	<0.001
	NO_2_	0.201 (0.136, 0.266)	<0.001	0.379 (0.309, 0.449)	<0.001	0.236 (0.168, 0.305)	<0.001
	O_3_	−0.099 (−0.141, −0.056)	<0.001	−0.267 (−0.307, −0.227)	<0.001	−0.090 (−0.132, −0.048)	<0.001
	PM_10_	0.124 (0.094, 0.153)	<0.001	0.143 (0.115, 0.172)	<0.001	0.139 (0.107, 0.172)	<0.001
	PM_2.5_	0.179 (0.138, 0.220)	<0.001	0.192 (0.157, 0.228)	<0.001	0.229 (0.182, 0.277)	<0.001
	SO_2_	0.192 (0.0141, 0.244)	<0.001	0.488 (0.411, 0.565)	<0.001	0.348 (0.279, 0.416)	<0.001
	APS	0.003 (0.003, 0.004)	<0.001	0.003 (0.002, 0.004)	<0.001	0.005 (0.004, 0.006)	<0.001

APS: Air Pollution Score. The model was adjusted for maternal age, ppBMI, ethnicity, mother’s education, gestational week, litter size, fetal category, infant weight, infant sex, history of hypertensive disorders or gestational diabetes mellitus, smoking status, alcohol use, season of delivery, comorbidities, and ambient temperature and humidity during pregnancy. Abbreviations: First trimester: early pregnancy; Second trimester: mid-pregnancy; Third trimester: late pregnancy.

**Table 3:** Generalized estimating equations for pollutants (per 1 μg/m^3^ for CO; and per 10 μg/m^3^ for NO_2_, O_3_, PM_10_, PM_2.5_, SO_2_) exposure and abnormal GWG and GWG.

Outcome	Exposure	β (95%CI)	*p*	Outcome	OR (95%CI)	*p*	Outcome	OR (95%CI)	*p*
GWG (kg)				GWG ^a^			GWG ^b^		
	CO	0.543 (1.408, 2.104)	<0.001		1.273 (1.113, 1.456)	<0.001		1.165 (0.994, 1.366)	0.059
	NO_2_	0.226 (1.206, 1.302)	<0.001		1.088 (1.059, 1.117)	<0.001		1.010 (0.978, 1.042)	0.559
	O_3_	−0.144 (0.846, 0.886)	<0.001		0.948 (0.936, 0.960)	<0.001		1.019 (1.004, 1.034)	0.014
	PM_10_	0.108 (1.094, 1.134)	<0.001		1.049 (1.036, 1.062)	<0.001		1.021 (1.006, 1.036)	0.006
	PM_2.5_	0.160 (1.145, 1.204)	<0.001		1.073 (1.055, 1.093)	<0.001		1.032 (1.011, 1.054)	0.003
	SO_2_	0.198 (1.179, 1.262)	<0.001		1.098 (1.075, 1.122)	<0.001		1.050 (1.024, 1.077)	<0.001

GWG ^a^: Excessive weight gain during pregnancy, using appropriate weight gain during pregnancy as a control. GWG ^b^: Insufficient weight gain during pregnancy, using appropriate weight gain during pregnancy as a control. The model was adjusted for maternal age, ppBMI, ethnicity, mother’s education, gestational week, litter size, fetal category, infant weight, infant sex, history of hypertensive disorders or gestational diabetes mellitus, smoking status, alcohol use, season of delivery, comorbidities, and ambient temperature and humidity during pregnancy.

## Data Availability

The data presented in this study are available on request from the corresponding author, as the data used in this study were obtained from hospital medical record systems. Due to ethical restrictions and participant privacy protection, the raw data cannot be made publicly available. For access to the raw data, please submit a reasonable request to the corresponding author.

## Supplementary Materials

The following supporting information can be downloaded at: https://www.mdpi.com/article/10.3390/toxics14030264/s1, Figure S1: Levels of exposure to air pollutants during pregnancy; Figure S2: Restricted cubic spline curves were used to analyze the association between pollutants and excessive GWG; Figure S3: Restricted cubic spline curves were used to analyze the association between pollutants and insufficient GWG; Table S1: Association between exposure to pollutants (per 1 μg/m^3^ for CO; and per 10 μg/m^3^ for NO_2,_ O_3_, PM_10_, PM_2.5_, SO_2_) and GWG pattern; Table S2: Association between pollutants (per 1 μg/m^3^ for CO; and per 10 μg/m^3^ for NO_2_, O_3_, PM_10_, PM_2.5_, SO_2_) exposure and GWG stratified by pre-pregnancy BMI; Table S3: Relationship between pollutants (per 1 μg/m^3^ for CO; and per 10 μg/m^3^ for NO_2_, O_3_, PM_10_, PM_2.5_, SO_2_) exposure and GWG stratified by maternal age; Table S4: Sensitivitly analysis of association between exposure to pollutants (per 1 μg/m^3^ for CO; and per 10 μg/m^3^ for NO_2_, O_3_, PM_10_, PM_2.5_, SO_2_) and GWG after excluding pregnant women less than 34 weeks of gestation; Table S5: Sensitivitly analysis of association between exposure to pollutants (per 1 μg/m^3^ for CO; and per 10 μg/m^3^ for NO_2_, O_3_, PM_10_, PM_2.5_, SO_2_) and GWG pattern after excluding pregnant women less than 34 weeks of gestation; Table S6: Sensitivity analyses of the association between exposure to pollutants (per 1 μg/m^3^ for CO; and per 10 μg/m^3^ for NO_2_, O_3_, PM_10_, PM_2.5_, SO_2_) and GWG after excluding stillbirths, stillbirths, induced deliveries, and multiparous pregnancies; Table S7: Sensitivity analyses of the association between exposure to pollutants (per 1 μg/m^3^ for CO; and per 10 μg/m^3^ for NO_2_, O_3_, PM_10_, PM_2.5_, SO_2_) and GWG pattern after excluding stillbirths, stillbirths, induced deliveries, and multiparous pregnancies; Table S8: Sensitivity analysis of the simplified model (excluding infant birth weight, gestational age, and pregnancy complications) examining the association between pollutant exposure (per 1 μg/m^3^ for CO; and per 10 μg/m^3^ for NO_2_, O_3_, PM_10_, PM_2.5_, SO_2_) and gestational weight gain; Table S9: Sensitivity analysis of the simplified model (excluding infant birth weight, gestational age, and pregnancy complications) examining the association between pollutant exposure(per 1 μg/m^3^ for CO; and per 10 μg/m^3^ for NO_2_, O_3_, PM_10_, PM_2.5_, SO_2_) and patterns of weight gain during pregnancy.

## Abbreviations

The following abbreviations are used in this manuscript:

APS	Air pollution score
ART	Assisted reproductive technology
BMI	Body Mass Index
CO	Carbon monoxide
CHAP	China High Air Pollution
GEE	Generalized Estimating Equation
GWG	Gestational Weight Gain
GDM	Gestational diabetes mellitus
IOM	International Organization for Migration
NO_2_	Nitrogen Dioxide
O_3_	Ozone
RCS	Restricted Cubic Spline
SO_2_	Sulfur dioxide
ppBMI	pre-pregnancy Body Mass Index
PM_10_	Particulate Matter with diameter ≤ 10 μm
PM_2.5_	Particulate Matter with diameter ≤ 2.5 μm
