# Supplementary material for: Associations Between Air Pollution Exposure and Gestational Weight Gain Pattern: Evidence from a Large-Scale Hospital-Based Retrospective Cohort Study
Source: Toxics. 2026 Mar 18;14(3):264. doi: 10.3390/toxics14030264 (PMC13030804; doi:10.3390/toxics14030264)
Supplement: Supplementary file 1 [file toxics-14-00264-s001.zip › toxics-4168210-supplementary.pdf]

Supplemental Material

Associations Between Air Pollution Exposure and Gestational Weight Gain Pattern: Evidence From a Large-Scale Hospital-Based Retrospective Cohort Study

Table of contents

Figure S1. Levels of exposure to air pollutants during pregnancy.

Figure S2. Restricted cubic spline curves were used to analyze the association between pollutants and excessive GWG.

Figure S3. Restricted cubic spline curves were used to analyze the association between pollutants and insufficient GWG.

Table S1. Association between exposure to pollutants (per 1 μg/m^3 for CO; and per 10 μg/m^3 for NO_2, O_3, PM_{10}, $PM_{2.5}$, SO_2) and GWG pattern.

Table S2. Association between pollutants (per 1 μg/m^3 for CO; and per 10 μg/m^3 for NO_2, O_3, PM_{10}, $PM_{2.5}$, SO_2) exposure and GWG stratified by pre-pregnancy BMI.

Table S3. Relationship between pollutants (per 1 μg/m^3 for CO; and per 10 μg/m^3 for NO_2, O_3, PM_{10}, $PM_{2.5}$, SO_2) exposure and GWG stratified by maternal age.

Table S4. Sensitivitly analysis of association between exposure to pollutants (per 1 μg/m^3 for CO; and per 10 μg/m^3 for NO_2, O_3, PM_{10}, $PM_{2.5}$, SO_2) and GWG after excluding pregnant women less than 34 weeks of gestation.

Table S5. Sensitivitly analysis of association between exposure to pollutants (per 1 μg/m^3 for CO; and per 10 μg/m^3 for NO_2, O_3, PM_{10}, $PM_{2.5}$, SO_2) and GWG pattern after excluding pregnant women less than 34 weeks of gestation.

Table S6. Sensitivity analyses of the association between exposure to pollutants (per 1 μg/m^3 for CO; and per 10 μg/m^3 for NO_2, O_3, PM_{10}, $PM_{2.5}$, SO_2) and GWG after excluding stillbirths, stillbirths, induced deliveries, and multiparous pregnancies.

Table S7. Sensitivity analyses of the association between exposure to pollutants (per 1 μg/m^3 for CO; and per 10 μg/m^3 for NO_2, O_3, PM_{10}, $PM_{2.5}$, SO_2) and GWG pattern after excluding stillbirths, stillbirths, induced deliveries, and multiparous pregnancies.

Table S8. Sensitivity analysis of the simplified model (excluding infant birth weight, gestational age, and pregnancy complications) examining the association between pollutant exposure (per 1 μg/m3 for CO; and per 10 μg/m3 for NO2, O3, PM10, PM2.5, SO2) and gestational weight gain.

Table S9. Sensitivity analysis of the simplified model (excluding infant birth weight, gestational age, and pregnancy complications) examining the association between pollutant exposure(per 1 μg/m3 for CO; and per 10 μg/m3 for NO2, O3, PM10, PM2.5, SO2) and patterns of weight gain during pregnancy.

Figure S1.

Levels of exposure to air pollutants during pregnancy.

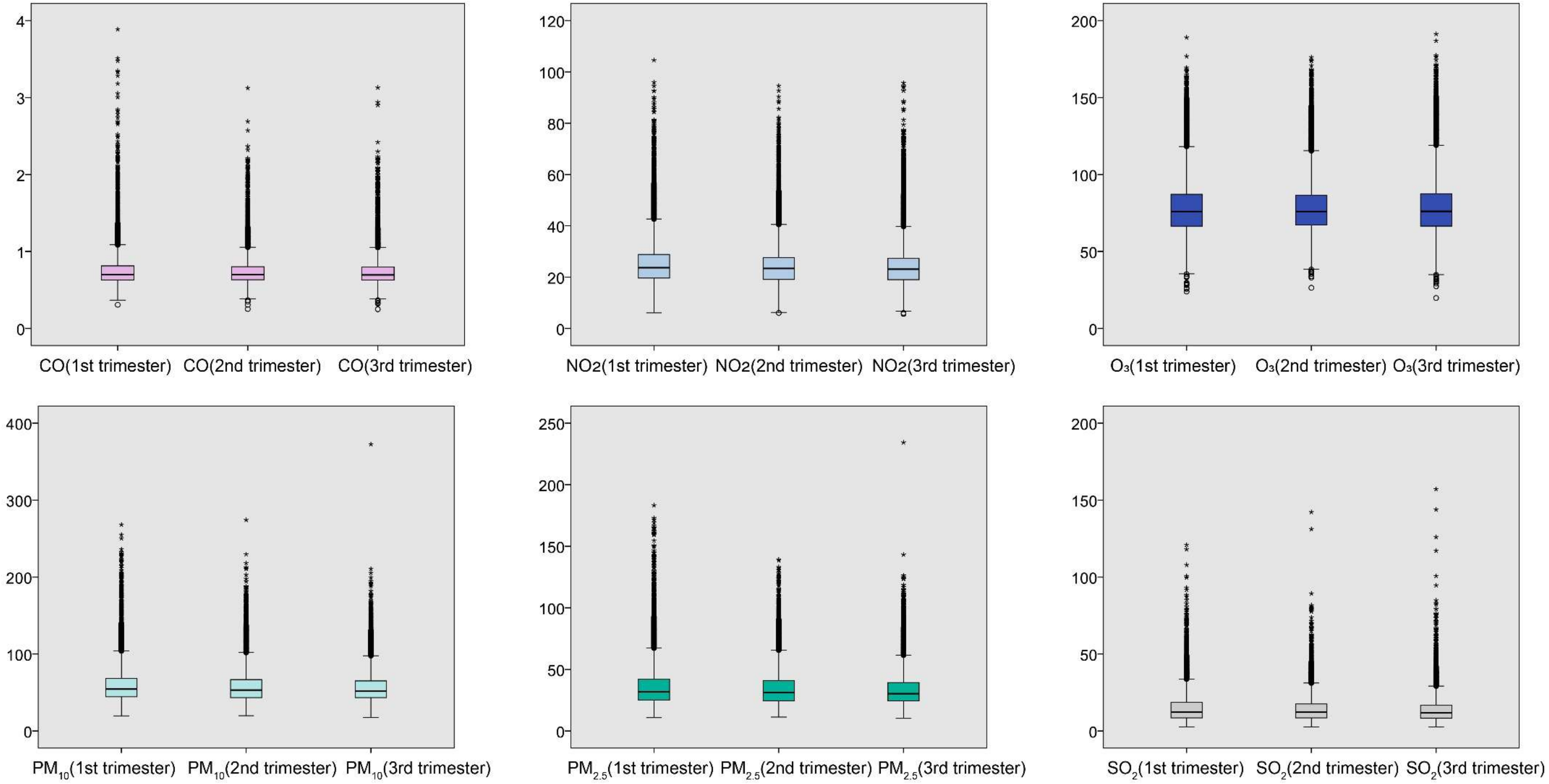

Figure S2. **Restricted cubic spline curves were used to analyze the association between pollutants($\mu g/m^3$) and excessive GWG.**

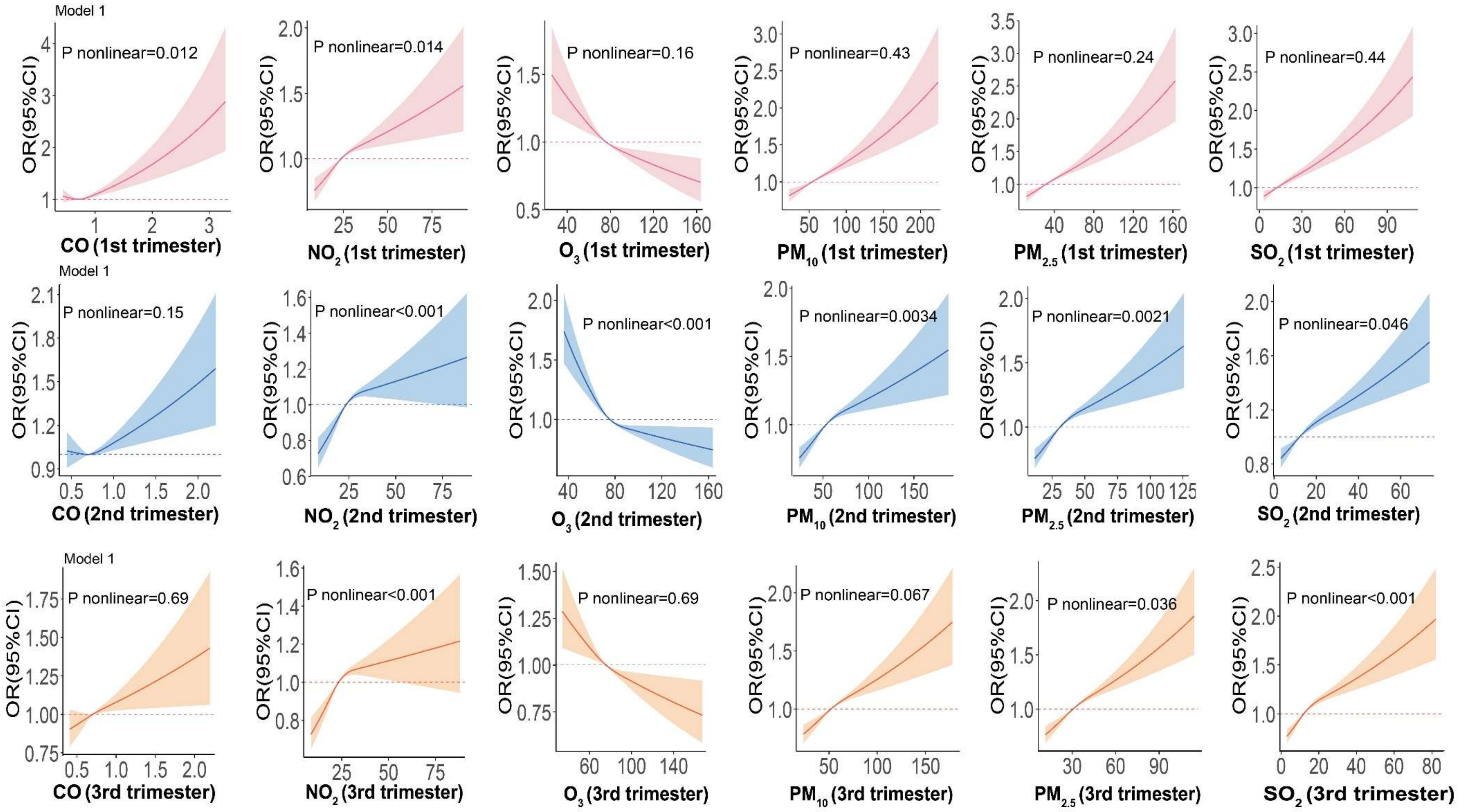

The model was adjusted for maternal age, ppBMI, ethnicity, mother's education, gestational week, litter size, fetal category, infant weight, infant sex, history of hypertensive disorders or gestational diabetes mellitus, smoking status, alcohol use, season of delivery, comorbidities, and ambient temperature and humidity during pregnancy. Abbreviations: 1st trimester, early pregnancy; 2nd trimester, mid-pregnancy; 3rd trimester, late pregnancy.

Figure S3. **Restricted cubic spline curves were used to analyze the association between pollutants(μg/m³) and insufficient GWG.**

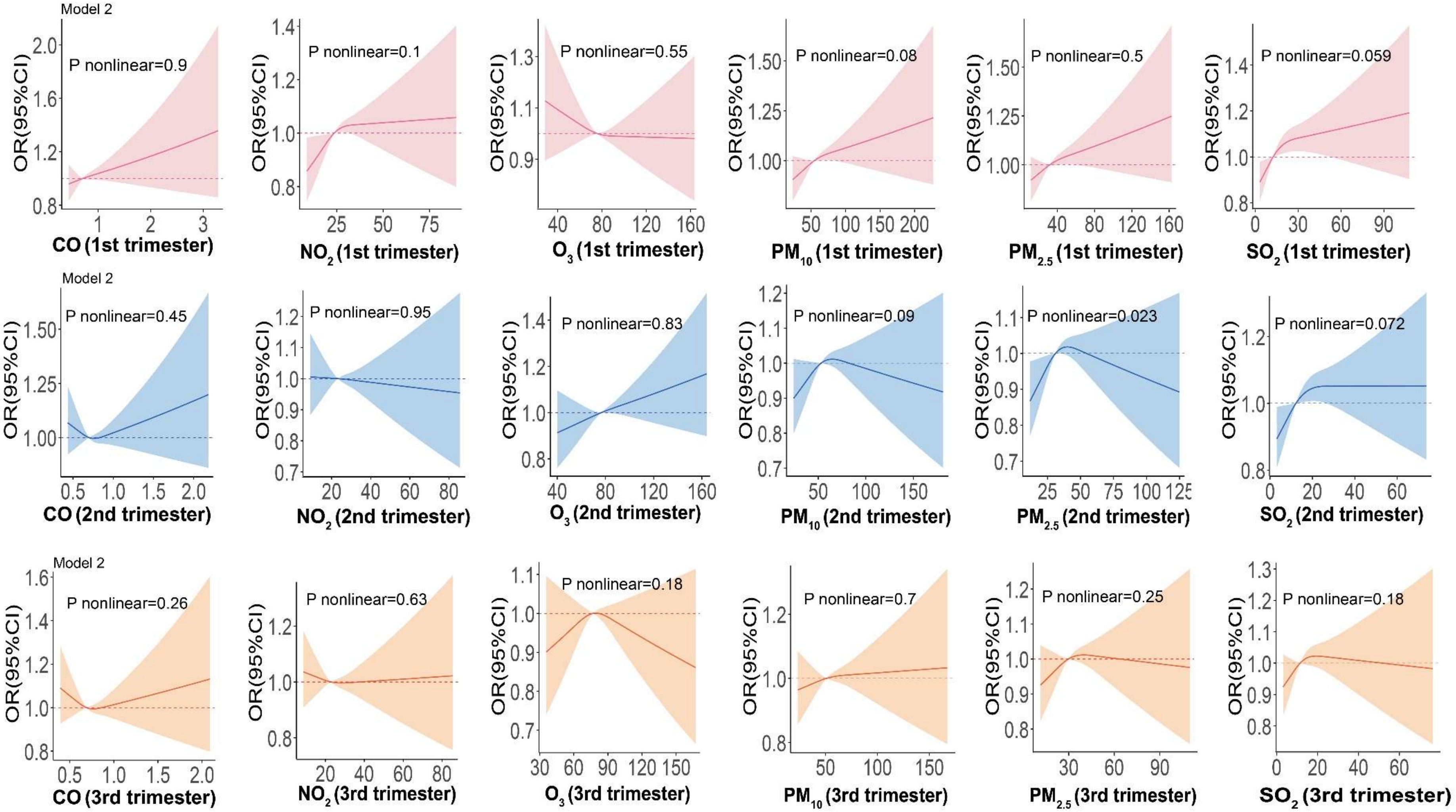

The model was adjusted for maternal age, ppBMI, ethnicity, mother's education, gestational week, litter size, fetal category, infant weight, infant sex, history of hypertensive disorders or gestational diabetes mellitus, smoking status, alcohol use, season of delivery, comorbidities, and ambient temperature and humidity during pregnancy. Abbreviations: 1st trimester, early pregnancy; 2nd trimester, mid-pregnancy; 3rd trimester, late pregnancy.

Table S1. Association between exposure to pollutants (per 1 μg/m^3 for CO; and per 10 μg/m^3 for NO_2, O_3, PM_{10}, $PM_{2.5}$, SO_2) and GWG pattern.

Outcomes	Exposures	First trimester		Second trimester		Third trimester	
		OR(95%CI)	P	OR(95%CI)	P	OR(95%CI)	P
GWG[a]							
	CO	1.377(1.201,1.578)	<0.001	1.271(1.086,1.488)	0.003	1.307(1.110,1.539)	0.001
	NO_2	1.098(1.068,1.130)	<0.001	1.088(1.056,1.120)	<0.001	1.084(1.053,1.116)	<0.001
	O_3	0.948(0.930,0.966)	<0.001	0.936(0.918,0.954)	<0.001	0.959(0.941,0.976)	<0.001
	PM_{10}	1.056(1.043,1.070)	<0.001	1.051(1.037,1.066)	<0.001	1.058(1.043,1.073)	<0.001
	$PM_{2.5}$	1.082(1.063,1.102)	<0.001	1.077(1.056,1.098)	<0.001	1.095(1.073,1.118)	<0.001
	SO_2	1.102(1.078,1.127)	<0.001	1.108(1.079,1.139)	<0.001	1.135(1.102,1.169)	<0.001
	APS	1.016(1.013,1.019)	<0.001	1.015(1.012,1.018)	<0.001	1.018(1.015,1.022)	<0.001
GWG[b]							
	CO	1.136(0.970,1.330)	0.115	1.061(0.878,1.282)	0.542	1.011(0.825,1.240)	0.914
	NO_2	1.032(0.998,1.067)	0.066	0.994(0.959,1.030)	0.741	0.996(0.961,1.033)	0.838
	O_3	0.990(0.967,1.013)	0.397	1.020(0.998,1.043)	0.080	0.996(0.975,1.018)	0.733
	PM_{10}	1.016(1.001,1.032)	0.033	1.005(0.989,1.021)	0.547	1.006(0.989,1.023)	0.490
	$PM_{2.5}$	1.022(1.001,1.043)	0.045	1.007(0.984,1.031)	0.533	1.009(0.983,1.034)	0.507
	SO_2	1.031(1.004,1.058)	0.023	1.027(0.993,1.061)	0.116	1.013(0.976,1.052)	0.502
	APS	1.002(0.998,1.005)	0.379	1.000(1.000,1.000)	0.138	1.001(0.993,1.009)	0.784

GWG[a] : Excessive weight gain during pregnancy, using appropriate weight gain during pregnancy as a control

GWG[b]: Insufficient weight gain during pregnancy, using appropriate weight gain during pregnancy as a control

APS: Air Pollution Scores

The model was adjusted for maternal age, ppBMI, ethnicity, mother's education, gestational week, litter size, fetal category, infant weight, infant sex, history of hypertensive disorders or gestational diabetes mellitus, smoking status, alcohol use, season of delivery, comorbidities, and ambient temperature and humidity during pregnancy.

Abbreviations: First trimester: early pregnancy; Second trimester: mid-pregnancy; Third trimester: late pregnancy.

Table S2. Association between pollutants (per 1 μg/m^3 for CO; and per 10 μg/m^3 for NO_2, O_3, PM_{10}, $PM_{2.5}$, SO_2) exposure and GWG stratified by pre-pregnancy BMI.

Outcomes	Exposures	Times	BMI < 24		BMI≥24		
			β(95%CI)	P	β(95%CI)	P	*P* for interaction
GWG(kg)	CO	First trimester	0.664(0.337,0.990)	<0.001	0.623(-0.391,1.638)	0.229	0.168
		Second trimester	0.767(0.381,1.153)	<0.001	1.095(-0.047,2.238)	0.060	0.468
		Third trimester	0.688(0.284,1.092)	0.001	1.709(0.534,2.884)	0.004	0.901
	NO_2	First trimester	0.203(0.134,0.271)	<0.001	0.271(0.073,0.469)	0.007	0.036
		Second trimester	0.322(0.251,0.394)	<0.001	0.375(0.170,0.579)	<0.001	0.324
		Third trimester	0.223(0.151,0.296)	<0.001	0.353(0.147,0.559)	0.001	0.312
	O_3	First trimester	-0.113 (-0.159,-0.068)	<0.001	-0.124(-0.250,0.002)	0.054	0.756
		Second trimester	-0.297(-0.340,-0.254)	<0.001	-0.291(-0.408,-0.174)	<0.001	0.757
		Third trimester	-0.106(-0.151,-0.061)	<0.001	-0.114(-0.241,0.014)	0.082	0.218
	PM_{10}	First trimester	0.118(0.087,0.149)	<0.001	0.193(0.101,0.286)	<0.001	<0.001
		Second trimester	0.121(0.088,0.155)	<0.001	0.204(0.106,0.302)	0.001	0.009

	Third trimester	0.133(0.099,0.167)	<0.001	0.224(0.124,0.323)	<0.001	0.072
$PM_{2.5}$	First trimester	0.178(0.135,0.222)	<0.001	0.253 (0.122,0.385)	<0.001	0.001
	Second trimester	0.180(0.133,0.228)	<0.001	0.295(0.153,0.437)	<0.001	0.026
	Third trimester	0.223(0.173,0.272)	<0.001	0.337 (0.190,0.484)	<0.001	<0.001
SO_2	First trimester	0.198(0.145,0.252)	<0.001	0.222(0.058,0.386)	0.008	0.174
	Second trimester	0.243 (0.178,0.308)	<0.001	0.350(0.155,0.545)	<0.001	0.352
	Third trimester	0.315(0.244,0.387)	<0.001	0.549(0.331,0.767)	<0.001	0.257

The model was adjusted for maternal age, ppBMI, ethnicity, mother's education, gestational week, litter size, fetal category, infant weight, infant sex, history of hypertensive disorders or gestational diabetes mellitus, smoking status, alcohol use, season of delivery, comorbidities, and ambient temperature and humidity during pregnancy.

Abbreviations: First trimester: early pregnancy; Second trimester: mid-pregnancy; Third trimester: late pregnancy.

Table S3. Relationship between pollutants (per 1 μg/m^3 for CO; and per 10 μg/m^3 for NO_2, O_3, PM_{10}, $PM_{2.5}$, SO_2) exposure and GWG stratified by maternal age.

Outcomes	Exposures	Times	Age < 30		Age≥30		
			β(95%CI)	P	β(95%CI)	P	*P* for interaction
GWG(kg)	CO	First trimester	0.670(0.261,1.079)	0.001	0.558(0.077,1.040)	0.023	0.055
		Second trimester	0.709(0.225,1.193)	0.004	0.907(0.350,1.463)	0.001	0.492
		Third trimester	0.759(0.248,1.270)	0.004	0.908(0.334,1.482)	0.002	0.739
	NO_2	First trimester	0.170(0.082,0.259)	<0.001	0.235(0.140,0.331)	<0.001	0.152
		Second trimester	0.293(0.200,0.386)	<0.001	0.346(0.248,0.444)	<0.001	0.748
		Third trimester	0.214(0.121,0.308)	<0.001	0.262(0.162,0.361)	<0.001	0.855
	O_3	First trimester	-0.103(-0.162,-0.044)	0.001	-0.088(-0.150,-0.026)	0.006	0.136
		Second trimester	-0.293(-0.348,-0.238)	<0.001	-0.241(-0.299,-0.183)	<0.001	0.203
		Third trimester	-0.108(-0.166,-0.050)	<0.001	-0.067(-0.128,-0.006)	0.032	0.071
	PM_{10}	First trimester	0.099(0.060,0.138)	<0.001	0.167(0.122,0.212)	<0.001	<0.001
		Second trimester	0.101(0.059,0.143)	<0.001	0.170(0.123,0.217)	<0.001	0.002
		Third trimester	0.118(0.074,0.161)	<0.001	0.174(0.125,0.222)	<0.001	0.053
	$PM_{2.5}$	First trimester	0.141(0.086,0.195)	<0.001	0.245(0.183,0.308)	<0.001	<0.001
		Second trimester	0.149(0.089,0.208)	<0.001	0.247(0.179,0.315)	<0.001	0.002
		Third trimester	0.187(0.125,0.250)	<0.001	0.293(0.222,0.364)	<0.001	0.028
	SO_2	First trimester	0.176(0.109,0.244)	<0.001	0.229(0.150,0.309)	<0.001	<0.001
		Second trimester	0.249(0.166,0.331)	<0.001	0.273 (0.174,0.371)	<0.001	0.081
		Third trimester	0.364(0.273,0.454)	<0.001	0.326(0.221,0.430)	<0.001	0.381

The model was adjusted for maternal age, ppBMI, ethnicity, mother's education, gestational week, litter size, fetal category, infant weight, infant sex, history of hypertensive disorders or gestational diabetes mellitus, smoking status, alcohol use, season of delivery, comorbidities, and ambient temperature and humidity during pregnancy.

Abbreviations: First trimester: early pregnancy; Second trimester: mid-pregnancy; Third trimester: late pregnancy.

Table S4. Sensitivitly analysis of association between exposure to pollutants (per 1 μg/m^3 for CO; and per 10 μg/m^3 for NO_2, O_3, PM_{10}, $PM_{2.5}$, SO_2) and GWG after excluding pregnant women less than 34 weeks of gestation.

Outcome	Exposures	First trimester		Second trimester		Third trimester	
		β(95%CI)	P	β(95%CI)	P	β(95%CI)	P
GWG(kg)							
	CO	0.604(0.290,0.917)	<0.001	0.741(0.373,1.109)	<0.001	0.773(0.386,1.160)	<0.001
	NO_2	0.199(0.134,0.265)	<0.001	0.314(0.246,0.382)	<0.001	0.227(0.158,0.296)	<0.001
	O_3	-0.102(-0.145,-0.058)	<0.001	-0.272(-0.313,-0.232)	<0.001	-0.089(-0.131,-0.046)	<0.001
	PM_{10}	0.125(0.095,0.154)	<0.001	0.127(0.095,0.158)	<0.001	0.138(0.105,0.170)	<0.001
	$PM_{2.5}$	0.179(0.138,0.221)	<0.001	0.185(0.140,0.230)	<0.001	0.227(0.180,0.275)	<0.001
	SO_2	0.191(0.139,0.242)	<0.001	0.256(0.193,0.320)	<0.001	0.357(0.288,0.426)	<0.001

The model was adjusted for maternal age, ppBMI, ethnicity, mother's education, gestational week, litter size, fetal category, infant weight, infant sex, history of hypertensive disorders or gestational diabetes mellitus, smoking status, alcohol use, season of delivery, comorbidities, and ambient temperature and humidity during pregnancy.

Abbreviations: First trimester: early pregnancy; Second trimester: mid-pregnancy; Third trimester: late pregnancy.

Table S5. Sensitivitly analysis of association between exposure to pollutants (per 1 μg/m^3 for CO; and per 10 μg/m^3 for NO_2, O_3, PM_{10}, $PM_{2.5}$, SO_2) and GWG pattern after excluding pregnant women less than 34 weeks of gestation.

Outcomes	Exposures	First trimester		Second trimester		Third trimester	
		OR(95%CI)	P	OR(95%CI)	P	OR(95%CI)	P
GWG[a]							
	CO	1.374(1.197,1.576)	<0.001	1.242(1.059,1.456)	0.008	1.275(1.080,1.505)	0.004

	NO_2	1.099(1.068,1.131)	<0.001	1.087(1.055,1.119)	<0.001	1.082(1.050,1.115)	<0.001
	O_3	0.947(0.929,0.965)	<0.001	0.936(0.918,0.954)	<0.001	0.961(0.943,0.979)	<0.001
	PM_{10}	1.057(1.043,1.071)	<0.001	1.051(1.037,1.066)	<0.001	1.059(1.044,1.074)	<0.001
	$PM_{2.5}$	1.083(1.063,1.103)	<0.001	1.077(1.056,1.098)	<0.001	1.098(1.075,1.121)	<0.001
	SO_2	1.103(1.078,1.128)	<0.001	1.108(1.078,1.139)	<0.001	1.136(1.103,1.171)	<0.001
GWG[b]							
	CO	1.152(0.981,1.353)	0.084	1.058(0.872,1.283)	0.57	1.020(0.828,1.257)	0.851
	NO_2	1.039(1.004,1.075)	0.027	0.999(0.964,1.036)	0.954	1.001(0.965,1.039)	0.942
	O_3	0.985(0.962,1.009)	0.224	1.018(0.995,1.042)	0.134	0.997(0.975,1.020)	0.805
	PM_{10}	1.020(1.005,1.036)	0.010	1.008(0.991,1.025)	0.362	1.011(0.993,1.029)	0.222
	$PM_{2.5}$	1.027(1.005,1.049)	0.015	1.011(0.988,1.036)	0.348	1.017(0.991,1.043)	0.214
	SO_2	1.036(1.008,1.064)	0.010	1.030(0.996,1.065)	0.089	1.013(0.974,1.053)	0.518

GWG[a] : Excessive weight gain during pregnancy, using appropriate weight gain during pregnancy as a control

GWG[b]: Insufficient weight gain during pregnancy, using appropriate weight gain during pregnancy as a control

The model was adjusted for maternal age, ppBMI, ethnicity, mother's education, gestational week, litter size, fetal category, infant weight, infant sex, history of hypertensive disorders or gestational diabetes mellitus, smoking status, alcohol use, season of delivery, comorbidities, and ambient temperature and humidity during pregnancy.

Abbreviations: First trimester: early pregnancy; Second trimester: mid-pregnancy; Third trimester: late pregnancy.

Table S6. Sensitivity analyses of the association between exposure to pollutants (per 1 μg/m^3 for CO; and per 10 μg/m^3 for NO_2, O_3, PM_{10}, $PM_{2.5}$, SO_2) and GWG after excluding stillbirths, stillbirths, induced deliveries, and multiparous pregnancies.

Outcome	Exposures	First trimester		Second trimester		Third trimester	
		β(95%CI)	P	β(95%CI)	P	β(95%CI)	P
GWG(kg)							

CO	0.627(0.312,0.942)	<0.001	0.794(0.424,1.164)	<0.001	0.806(0.419,1.194)	<0.001
NO_2	0.199(0.134,0.265)	<0.001	0.314(0.245,0.382)	<0.001	0.221(0.152,0.290)	<0.001
O_3	-0.097(-0.141,-0.054)	<0.001	-0.270(-0.310,-0.229)	<0.001	-0.085(-0.128,-0.042)	<0.001
PM_{10}	0.124(0.094,0.154)	<0.001	0.124(0.092,0.156)	<0.001	0.132(0.099,0.165)	<0.001
$PM_{2.5}$	0.177(0.135,0.219)	<0.001	0.181(0.136,0.227)	<0.001	0.219(0.171,0.267)	<0.001
SO_2	0.186(0.134,0.238)	<0.001	0.254(0.189,0.318)	<0.001	0.342(0.273,0.411)	<0.001

The model was adjusted for maternal age, ppBMI, ethnicity, mother's education, gestational week, litter size, fetal category, infant weight, infant sex, history of hypertensive disorders or gestational diabetes mellitus, smoking status, alcohol use, season of delivery, comorbidities, and ambient temperature and humidity during pregnancy.

Abbreviations: First trimester: early pregnancy; Second trimester: mid-pregnancy; Third trimester: late pregnancy.

Table S7. Sensitivity analyses of the association between exposure to pollutants (per 1 μg/m^3 for CO; and per 10 μg/m^3 for NO_2, O_3, PM_{10}, $PM_{2.5}$, SO_2) and GWG pattern after excluding stillbirths, stillbirths, induced deliveries, and multiparous pregnancies.

Outcomes	Exposures	First trimester		Second trimester		Third trimester	
		OR(95%CI)	P	OR(95%CI)	P	OR(95%CI)	P
GWG[a]							
	CO	1.391(1.211,1.597)	<0.001	1.283(1.094,1.506)	0.002	1.301(1.101,1.537)	0.002
	NO_2	1.101(1.070,1.133)	<0.001	1.089(1.057,1.122)	<0.001	1.085(1.053,1.118)	<0.001
	O_3	0.950(0.932,0.969)	<0.001	0.939(0.920,0.957)	<0.001	0.962(0.944,0.980)	<0.001
	PM_{10}	1.058(1.044,1.072)	<0.001	1.052(1.038,1.067)	<0.001	1.059(1.044,1.074)	<0.001
	$PM_{2.5}$	1.084(1.064,1.104)	<0.001	1.078(1.057,1.100)	<0.001	1.096(1.074,1.119)	<0.001
	SO_2	1.101(1.076,1.126)	<0.001	1.106(1.076,1.138)	<0.001	1.133(1.100,1.167)	<0.001
GWG[b]							
	CO	1.144(0.975,1.342)	0.099	1.056(0.872,1.280)	0.576	1.012(0.822,1.245)	0.911
	NO_2	1.036(1.001,1.072)	0.041	0.998(0.963,1.035)	0.922	1.002(0.966,1.039)	0.924

O_3	0.991(0.968,1.015)	0.479	1.022(0.998,1.045)	0.068	0.998(0.976,1.020)	0.860
PM_{10}	1.018(1.002,1.033)	0.025	1.006(0.989,1.022)	0.495	1.009(0.991,1.026)	0.322
$PM_{2.5}$	1.023(1.001,1.045)	0.038	1.009(0.985,1.033)	0.480	1.012(0.987,1.038)	0.354
SO_2	1.031(1.004,1.059)	0.025	1.023(0.989,1.058)	0.183	1.009(0.971,1.049)	0.634

GWG[a] : Excessive weight gain during pregnancy, using appropriate weight gain during pregnancy as a control

GWG[b]: Insufficient weight gain during pregnancy, using appropriate weight gain during pregnancy as a control

The model was adjusted for maternal age, ppBMI, ethnicity, mother's education, gestational week, litter size, fetal category, infant weight, infant sex, history of hypertensive disorders or gestational diabetes mellitus, smoking status, alcohol use, season of delivery, comorbidities, and ambient temperature and humidity during pregnancy.

Abbreviations: First trimester: early pregnancy; Second trimester: mid-pregnancy; Third trimester: late pregnancy.

Table S8. Sensitivity analysis of the simplified model (excluding infant birth weight, gestational age, and pregnancy complications) examining the association between pollutant exposure (per 1 μg/m^3 for CO; and per 10 μg/m^3 for NO_2, O_3, PM_{10}, $PM_{2.5}$, SO_2) and gestational weight gain.

Outcome	Exposures	First trimester		Second trimester		Third trimester	
		β(95%CI)	P	β(95%CI)	P	β(95%CI)	P
GWG(kg)							
	CO	0.757(0.433,1.080)	<0.001	0.892(0.513,1.272)	<0.001	0.937(0.540,1.334)	<0.001
	NO_2	0.259(0.191,0.326)	<0.001	0.370(0.300,0.440)	<0.001	0.292(0.221,0.363)	<0.001
	O_3	-0.124(-0.168,-0.079)	<0.001	-0.303(-0.344,-0.261)	<0.001	-0.137(-0.181,-0.094)	<0.001
	PM_{10}	0.144(0.114,0.174)	<0.001	0.144(0.112,0.176)	<0.001	0.156(0.123,0.189)	<0.001
	$PM_{2.5}$	0.204(0.162,0.246)	<0.001	0.206(0.161,0.252)	<0.001	0.242(0.194,0.290)	<0.001
	SO_2	0.191(0.138,0.244)	<0.001	0.246(0.180,0.311)	<0.001	0.320(0.250,0.390)	<0.001

The model was adjusted for maternal age, ppBMI, ethnicity, mother's education , litter size, fetal category, infant sex, smoking status, alcohol use, season of delivery, and ambient temperature and humidity during pregnancy.

Abbreviations: First trimester: early pregnancy; Second trimester: mid-pregnancy; Third trimester: late pregnancy.

Table S9. Sensitivity analysis of the simplified model (excluding infant birth weight, gestational age, and pregnancy complications) examining the association between pollutant exposure(per 1 μg/m^3 for CO; and per 10 μg/m^3 for NO_2, O_3, PM_{10}, $PM_{2.5}$, SO_2) and patterns of weight gain during pregnancy.

Outcomes	Exposures	First trimester		Second trimester		Third trimester	
		OR(95%CI)	P	OR(95%CI)	P	OR(95%CI)	P
GWG[a]							
	CO	1.423(1.244,1.626)	<0.001	1.319(1.130,1.540)	<0.001	1.340(1.141,1.574)	<0.001
	NO_2	1.110(1.079,1.141)	<0.001	1.098(1.067,1.130)	<0.001	1.095(1.064,1.127)	<0.001
	O_3	0.947(0.929,0.964)	<0.001	0.933(0.916,0.951)	<0.001	0.952(0.935,0.970)	<0.001
	PM_{10}	1.060(1.046,1.073)	<0.001	1.055(1.041,1.069)	<0.001	1.061(1.047,1.076)	<0.001
	$PM_{2.5}$	1.087(1.068,1.106)	<0.001	1.082(1.062,1.102)	<0.001	1.098(1.076,1.119)	<0.001
	SO_2	1.101(1.077,1.125)	<0.001	1.108(1.079,1.138)	<0.001	1.130(1.098,1.163)	<0.001
GWG[b]							
	CO	1.108(0.949,1.293)	0.196	1.038(0.863,1.249)	0.691	0.981(0.804,1.197)	0.852
	NO_2	1.017(0.984,1.050)	0.318	0.984(0.951,1.018)	0.357	0.984(0.950,1.019)	0.359
	O_3	0.997(0.975,1.020)	0.820	1.025(1.003,1.047)	0.028	1.007(0.987,1.029)	0.487
	PM_{10}	1.011(0.997,1.026)	0.128	1.001(0.986,1.017)	0.855	1.003(0.987,1.020)	0.714
	$PM_{2.5}$	1.016(0.995,1.036)	0.129	1.004(0.981,1.027)	0.744	1.008(0.984,1.033)	0.496
	SO_2	1.034(1.008,1.060)	0.010	1.033(1.000,1.066)	0.047	1.027(0.990,1.065)	0.153

GWG[a] : Excessive weight gain during pregnancy, using appropriate weight gain during pregnancy as a control

GWG[b]: Insufficient weight gain during pregnancy, using appropriate weight gain during pregnancy as a control

The model was adjusted for maternal age, ppBMI, ethnicity, mother's education , litter size, fetal category, infant sex, smoking status, alcohol use, season of delivery, and ambient temperature and humidity during pregnancy.

Abbreviations: First trimester: early pregnancy; Second trimester: mid-pregnancy; Third trimester: late pregnancy.
